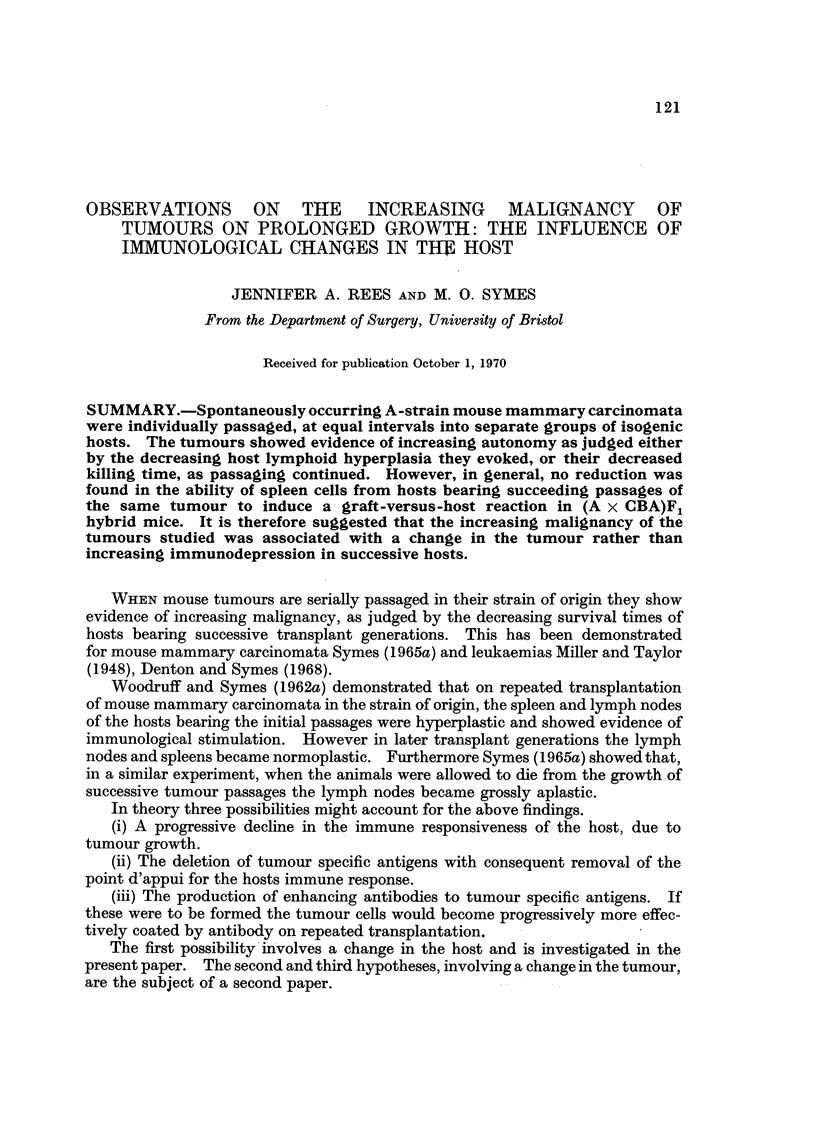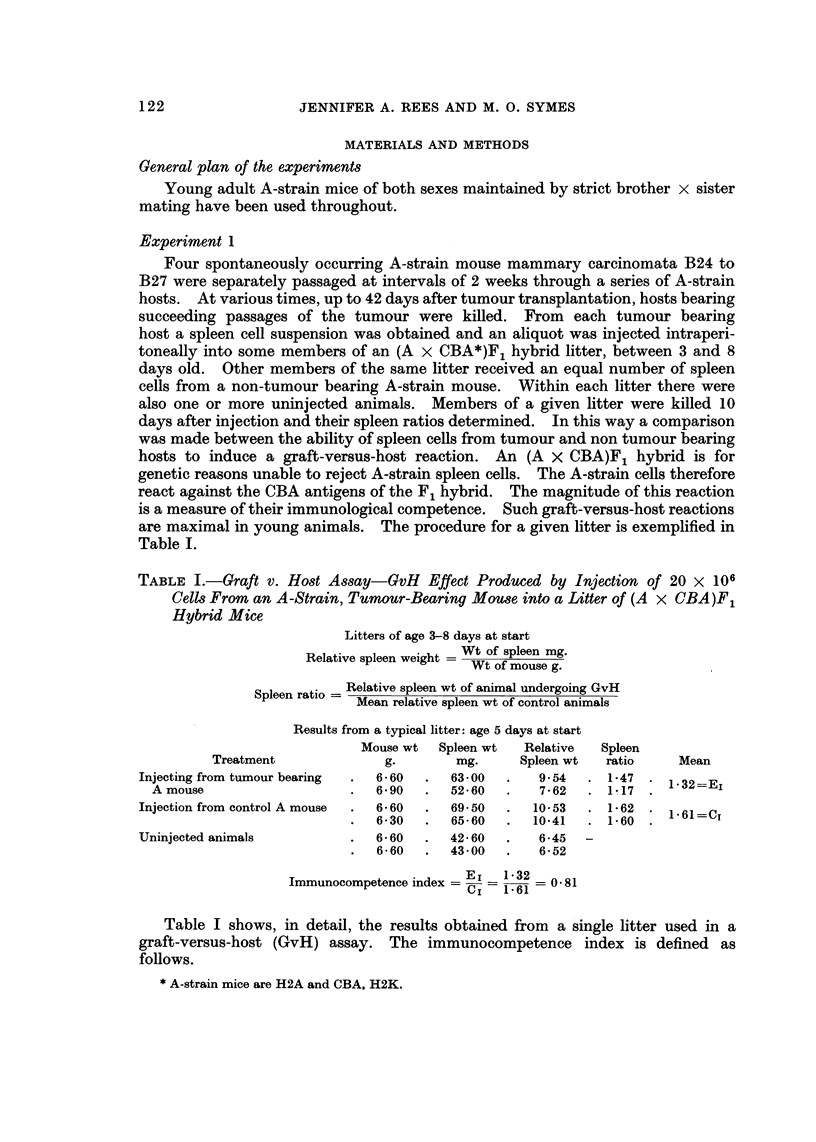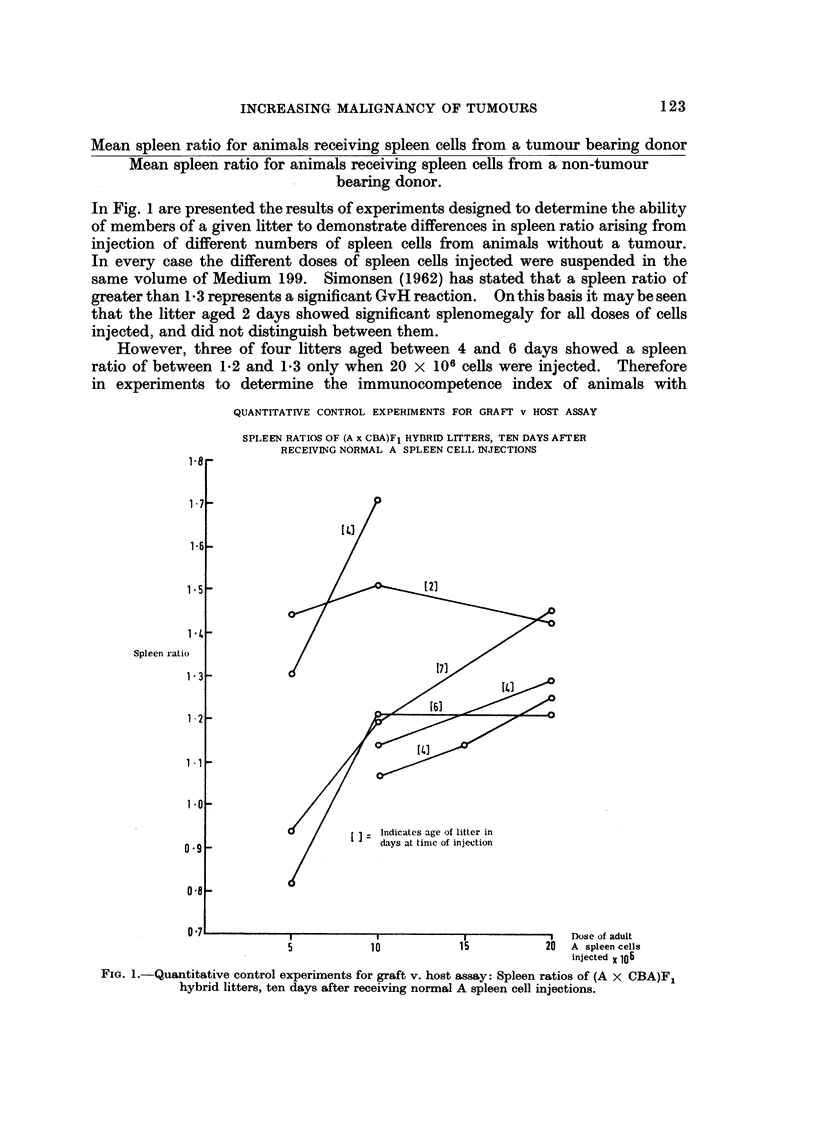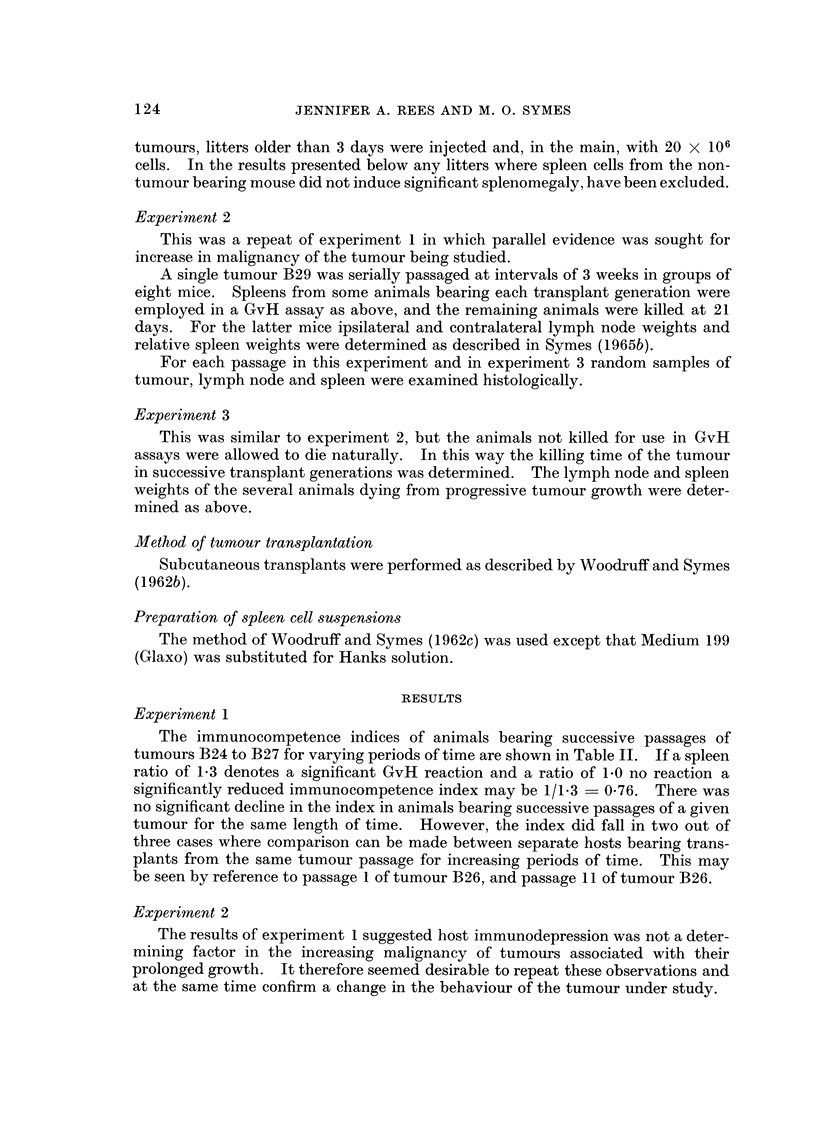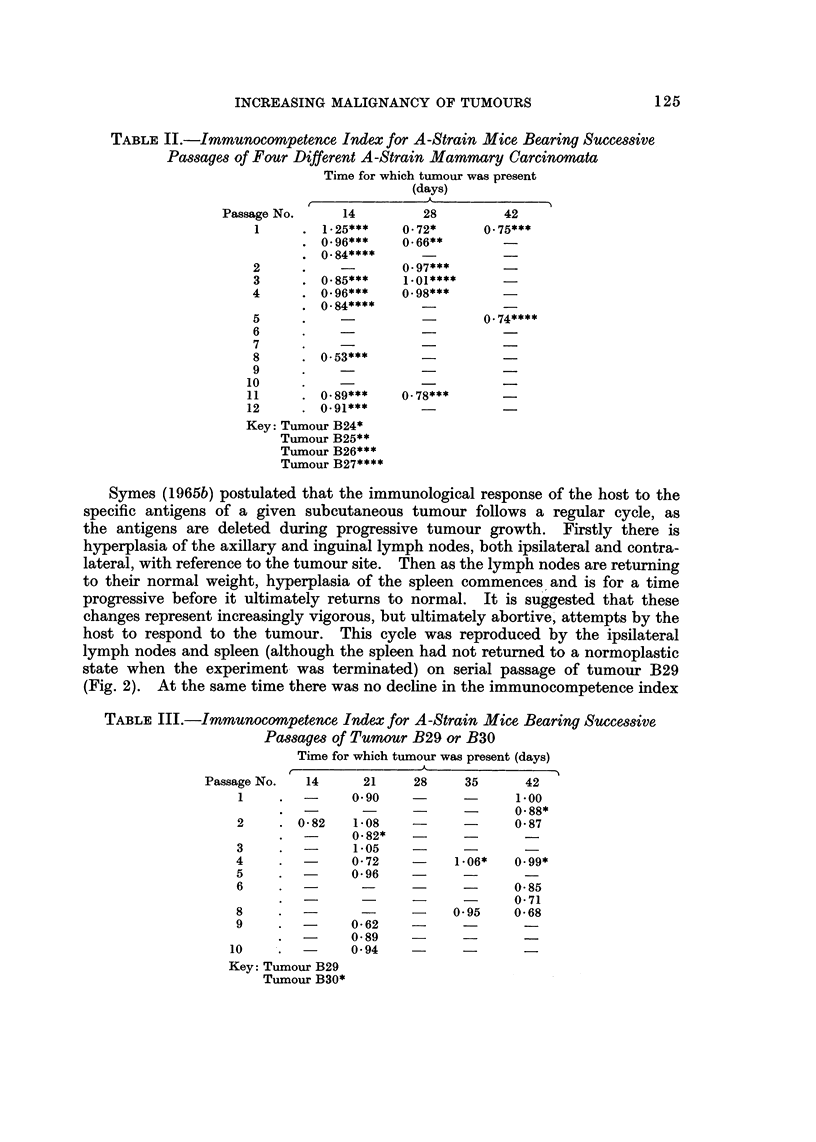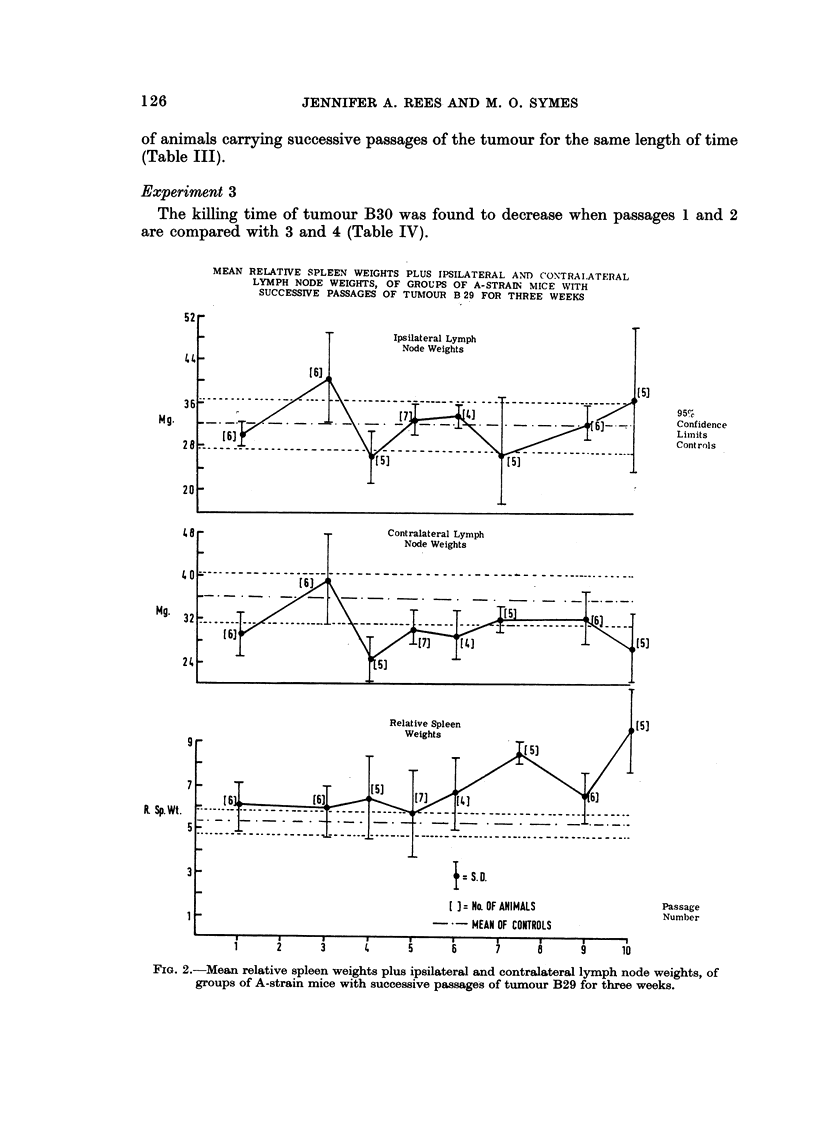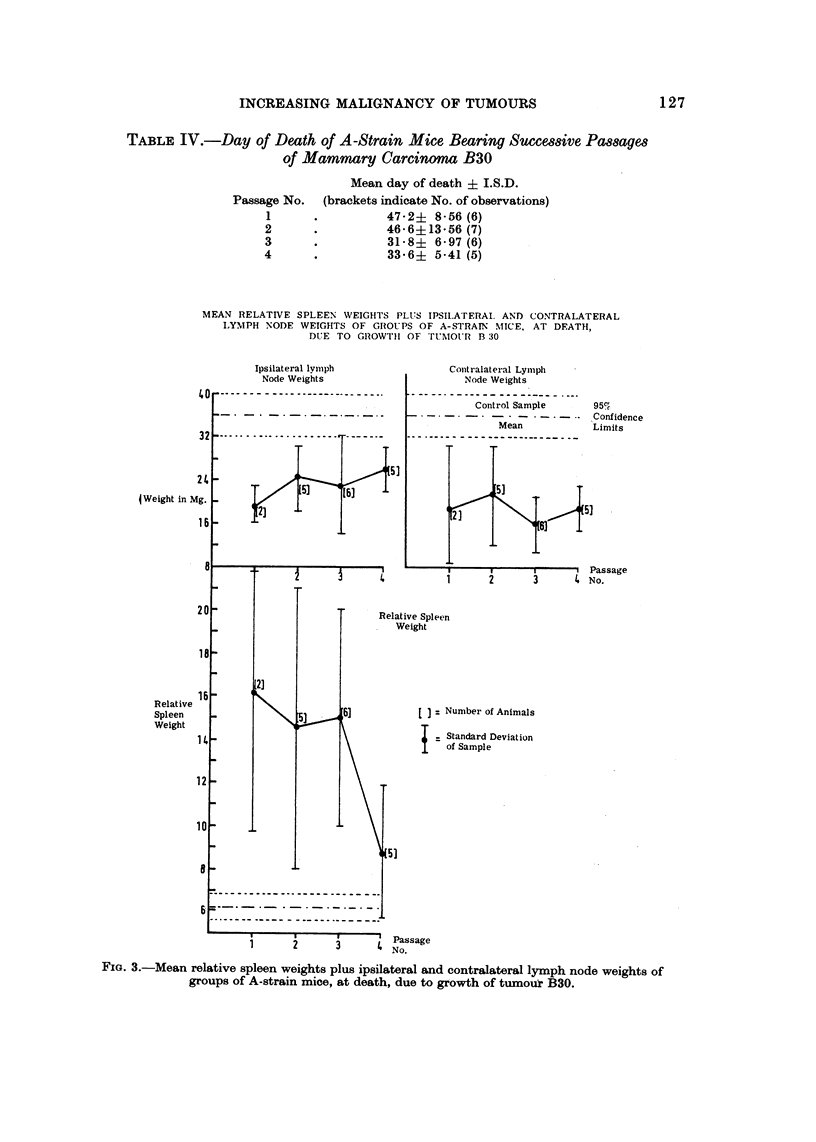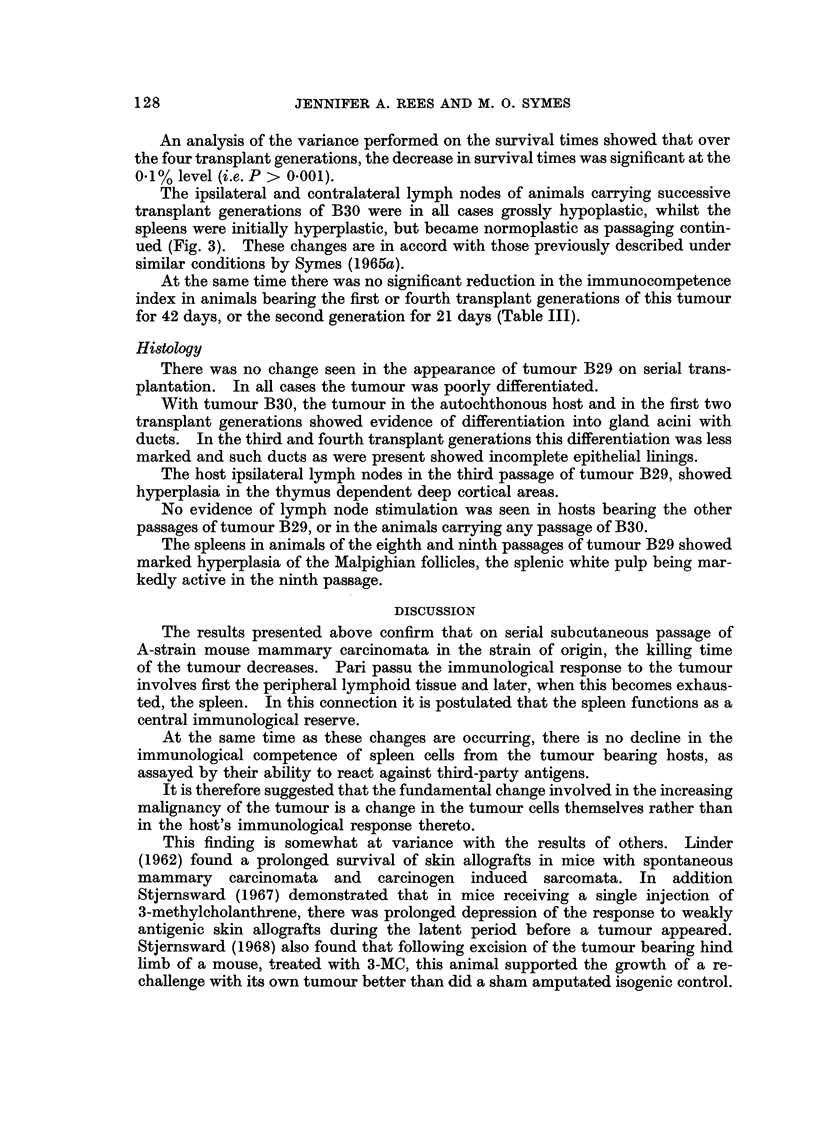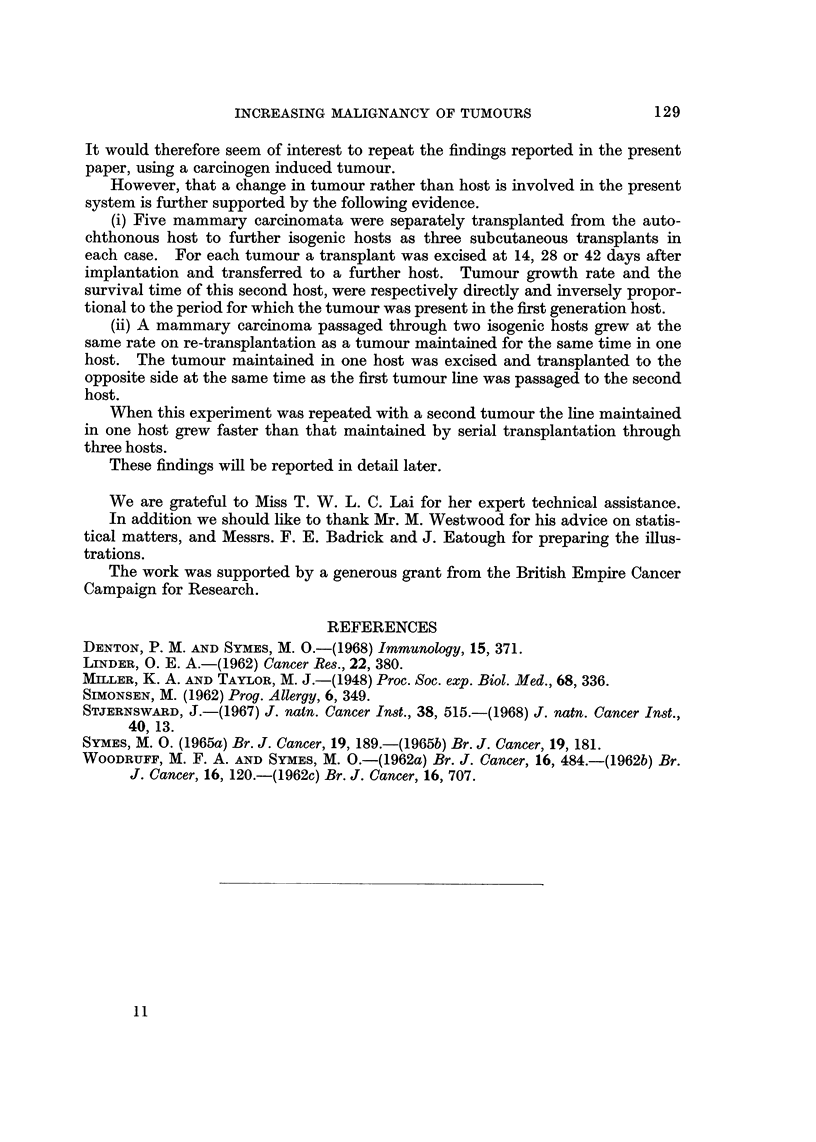# Observations on the Increasing Malignancy of Tumours on Prolonged Growth: The Influence of Immunological Changes in the Host

**DOI:** 10.1038/bjc.1971.17

**Published:** 1971-03

**Authors:** Jennifer A. Rees, M. O. Symes

## Abstract

Spontaneously occurring A-strain mouse mammary carcinomata were individually passaged, at equal intervals into separate groups of isogenic hosts. The tumours showed evidence of increasing autonomy as judged either by the decreasing host lymphoid hyperplasia they evoked, or their decreased killing time, as passaging continued. However, in general, no reduction was found in the ability of spleen cells from hosts bearing succeeding passages of the same tumour to induce a graft-versus-host reaction in (A × CBA)F_1_ hybrid mice. It is therefore suggested that the increasing malignancy of the tumours studied was associated with a change in the tumour rather than increasing immunodepression in successive hosts.


### OBSERVATIONS ON THE INCREASING MALIGNANCY OF TUMOURS ON PROLONGED GROWTH: THE INFLUENCE OF IMMUNOLOGICAL CHANGES IN THE HOST

JENNIFER A. REES AND M. O. SYMES

*From the Department of Surgery, University of Bristol*

Received for publication October 1, 1970

**SUMMARY.—Spontaneously occurring A-strain mouse mammary carcinomata were individually passaged, at equal intervals into separate groups of isogenic hosts. The tumours showed evidence of increasing autonomy as judged either by the decreasing host lymphoid hyperplasia they evoked, or their decreased killing time, as passaging continued. However, in general, no reduction was found in the ability of spleen cells from hosts bearing succeeding passages of the same tumour to induce a graft-versus-host reaction in (A $\times$ CBA)$F_1$ hybrid mice. It is therefore suggested that the increasing malignancy of the tumours studied was associated with a change in the tumour rather than increasing immunodepression in successive hosts.**

WHEN mouse tumours are serially passaged in their strain of origin they show evidence of increasing malignancy, as judged by the decreasing survival times of hosts bearing successive transplant generations. This has been demonstrated for mouse mammary carcinomata Symes (1965*a*) and leukaemias Miller and Taylor (1948), Denton and Symes (1968).

Woodruff and Symes (1962*a*) demonstrated that on repeated transplantation of mouse mammary carcinomata in the strain of origin, the spleen and lymph nodes of the hosts bearing the initial passages were hyperplastic and showed evidence of immunological stimulation. However in later transplant generations the lymph nodes and spleens became normoplastic. Furthermore Symes (1965*a*) showed that, in a similar experiment, when the animals were allowed to die from the growth of successive tumour passages the lymph nodes became grossly aplastic.

In theory three possibilities might account for the above findings.

(i) A progressive decline in the immune responsiveness of the host, due to tumour growth.

(ii) The deletion of tumour specific antigens with consequent removal of the point d'appui for the hosts immune response.

(iii) The production of enhancing antibodies to tumour specific antigens. If these were to be formed the tumour cells would become progressively more effectively coated by antibody on repeated transplantation.

The first possibility involves a change in the host and is investigated in the present paper. The second and third hypotheses, involving a change in the tumour, are the subject of a second paper.

### MATERIALS AND METHODS

*General plan of the experiments*

Young adult A-strain mice of both sexes maintained by strict brother × sister mating have been used throughout.

*Experiment* 1

Four spontaneously occurring A-strain mouse mammary carcinomata B24 to B27 were separately passaged at intervals of 2 weeks through a series of A-strain hosts. At various times, up to 42 days after tumour transplantation, hosts bearing succeeding passages of the tumour were killed. From each tumour bearing host a spleen cell suspension was obtained and an aliquot was injected intraperitoneally into some members of an (A × CBA*)$F_1$ hybrid litter, between 3 and 8 days old. Other members of the same litter received an equal number of spleen cells from a non-tumour bearing A-strain mouse. Within each litter there were also one or more uninjected animals. Members of a given litter were killed 10 days after injection and their spleen ratios determined. In this way a comparison was made between the ability of spleen cells from tumour and non tumour bearing hosts to induce a graft-versus-host reaction. An (A × CBA)$F_1$ hybrid is for genetic reasons unable to reject A-strain spleen cells. The A-strain cells therefore react against the CBA antigens of the $F_1$ hybrid. The magnitude of this reaction is a measure of their immunological competence. Such graft-versus-host reactions are maximal in young animals. The procedure for a given litter is exemplified in Table I.

TABLE I.—*Graft v. Host Assay—GvH Effect Produced by Injection of* 20 × $10^6$ *Cells From an A-Strain, Tumour-Bearing Mouse into a Litter of* (*A* × *CBA*)$F_1$ *Hybrid Mice*

Litters of age 3–8 days at start

$$\text{Relative spleen weight} = \frac{\text{Wt of spleen mg.}}{\text{Wt of mouse g.}}$$

$$\text{Spleen ratio} = \frac{\text{Relative spleen wt of animal undergoing GvH}}{\text{Mean relative spleen wt of control animals}}$$

Results from a typical litter: age 5 days at start

| Treatment | Mouse wt g. | Spleen wt mg. | Relative Spleen wt | Spleen ratio | Mean |
|---|---|---|---|---|---|
| Injecting from tumour bearing A mouse | 6·60 | 63·00 | 9·54 | 1·47 | $1·32 = E_I$ |
| | 6·90 | 52·60 | 7·62 | 1·17 | |
| Injection from control A mouse | 6·60 | 69·50 | 10·53 | 1·62 | $1·61 = C_I$ |
| | 6·30 | 65·60 | 10·41 | 1·60 | |
| Uninjected animals | 6·60 | 42·60 | 6·45 | – | |
| | 6·60 | 43·00 | 6·52 | | |

$$\text{Immunocompetence index} = \frac{E_I}{C_I} = \frac{1·32}{1·61} = 0·81$$

Table I shows, in detail, the results obtained from a single litter used in a graft-versus-host (GvH) assay. The immunocompetence index is defined as follows.

* A-strain mice are H2A and CBA, H2K.

$$\frac{\text{Mean spleen ratio for animals receiving spleen cells from a tumour bearing donor}}{\text{Mean spleen ratio for animals receiving spleen cells from a non-tumour bearing donor.}}$$

In Fig. 1 are presented the results of experiments designed to determine the ability of members of a given litter to demonstrate differences in spleen ratio arising from injection of different numbers of spleen cells from animals without a tumour. In every case the different doses of spleen cells injected were suspended in the same volume of Medium 199. Simonsen (1962) has stated that a spleen ratio of greater than 1·3 represents a significant GvH reaction. On this basis it may be seen that the litter aged 2 days showed significant splenomegaly for all doses of cells injected, and did not distinguish between them.

However, three of four litters aged between 4 and 6 days showed a spleen ratio of between 1·2 and 1·3 only when 20 × $10^6$ cells were injected. Therefore in experiments to determine the immunocompetence index of animals with

FIG. 1.—Quantitative control experiments for graft v. host assay: Spleen ratios of (A × CBA)$F_1$ hybrid litters, ten days after receiving normal A spleen cell injections.

tumours, litters older than 3 days were injected and, in the main, with $20 \times 10^6$ cells. In the results presented below any litters where spleen cells from the non-tumour bearing mouse did not induce significant splenomegaly, have been excluded.

*Experiment* 2

This was a repeat of experiment 1 in which parallel evidence was sought for increase in malignancy of the tumour being studied.

A single tumour B29 was serially passaged at intervals of 3 weeks in groups of eight mice. Spleens from some animals bearing each transplant generation were employed in a GvH assay as above, and the remaining animals were killed at 21 days. For the latter mice ipsilateral and contralateral lymph node weights and relative spleen weights were determined as described in Symes (1965*b*).

For each passage in this experiment and in experiment 3 random samples of tumour, lymph node and spleen were examined histologically.

*Experiment* 3

This was similar to experiment 2, but the animals not killed for use in GvH assays were allowed to die naturally. In this way the killing time of the tumour in successive transplant generations was determined. The lymph node and spleen weights of the several animals dying from progressive tumour growth were determined as above.

*Method of tumour transplantation*

Subcutaneous transplants were performed as described by Woodruff and Symes (1962*b*).

*Preparation of spleen cell suspensions*

The method of Woodruff and Symes (1962*c*) was used except that Medium 199 (Glaxo) was substituted for Hanks solution.

### RESULTS

*Experiment* 1

The immunocompetence indices of animals bearing successive passages of tumours B24 to B27 for varying periods of time are shown in Table II. If a spleen ratio of 1·3 denotes a significant GvH reaction and a ratio of 1·0 no reaction a significantly reduced immunocompetence index may be $1/1{\cdot}3 = 0{\cdot}76$. There was no significant decline in the index in animals bearing successive passages of a given tumour for the same length of time. However, the index did fall in two out of three cases where comparison can be made between separate hosts bearing transplants from the same tumour passage for increasing periods of time. This may be seen by reference to passage 1 of tumour B26, and passage 11 of tumour B26.

*Experiment* 2

The results of experiment 1 suggested host immunodepression was not a determining factor in the increasing malignancy of tumours associated with their prolonged growth. It therefore seemed desirable to repeat these observations and at the same time confirm a change in the behaviour of the tumour under study.

TABLE II.—*Immunocompetence Index for A-Strain Mice Bearing Successive Passages of Four Different A-Strain Mammary Carcinomata*

| Passage No. | Time for which tumour was present (days) 14 | 28 | 42 |
|---|---|---|---|
| 1 | 1·25*** | 0·72* | 0·75*** |
| | 0·96*** | 0·66** | — |
| | 0·84**** | — | — |
| 2 | — | 0·97*** | — |
| 3 | 0·85*** | 1·01**** | — |
| 4 | 0·96*** | 0·98*** | — |
| | 0·84**** | — | — |
| 5 | — | — | 0·74**** |
| 6 | — | — | — |
| 7 | — | — | — |
| 8 | 0·53*** | — | — |
| 9 | — | — | — |
| 10 | — | — | — |
| 11 | 0·89*** | 0·78*** | — |
| 12 | 0·91*** | — | — |

Key: Tumour B24*
Tumour B25**
Tumour B26***
Tumour B27****

Symes (1965*b*) postulated that the immunological response of the host to the specific antigens of a given subcutaneous tumour follows a regular cycle, as the antigens are deleted during progressive tumour growth. Firstly there is hyperplasia of the axillary and inguinal lymph nodes, both ipsilateral and contralateral, with reference to the tumour site. Then as the lymph nodes are returning to their normal weight, hyperplasia of the spleen commences and is for a time progressive before it ultimately returns to normal. It is suggested that these changes represent increasingly vigorous, but ultimately abortive, attempts by the host to respond to the tumour. This cycle was reproduced by the ipsilateral lymph nodes and spleen (although the spleen had not returned to a normoplastic state when the experiment was terminated) on serial passage of tumour B29 (Fig. 2). At the same time there was no decline in the immunocompetence index

TABLE III.—*Immunocompetence Index for A-Strain Mice Bearing Successive Passages of Tumour B29 or B30*

| Passage No. | Time for which tumour was present (days) 14 | 21 | 28 | 35 | 42 |
|---|---|---|---|---|---|
| 1 | — | 0·90 | — | — | 1·00 |
| | — | — | — | — | 0·88* |
| 2 | 0·82 | 1·08 | — | — | 0·87 |
| | — | 0·82* | — | — | — |
| 3 | — | 1·05 | — | — | — |
| 4 | — | 0·72 | — | 1·06* | 0·99* |
| 5 | — | 0·96 | — | — | — |
| 6 | — | — | — | — | 0·85 |
| | — | — | — | — | 0·71 |
| 8 | — | — | — | 0·95 | 0·68 |
| 9 | — | 0·62 | — | — | — |
| | — | 0·89 | — | — | — |
| 10 | — | 0·94 | — | — | — |

Key: Tumour B29
Tumour B30*

of animals carrying successive passages of the tumour for the same length of time (Table III).

*Experiment* 3

The killing time of tumour B30 was found to decrease when passages 1 and 2 are compared with 3 and 4 (Table IV).

FIG. 2.—Mean relative spleen weights plus ipsilateral and contralateral lymph node weights, of groups of A-strain mice with successive passages of tumour B29 for three weeks.

TABLE IV.—*Day of Death of A-Strain Mice Bearing Successive Passages of Mammary Carcinoma B30*

| Passage No. | Mean day of death ± I.S.D. (brackets indicate No. of observations) |
|---|---|
| 1 | 47·2± 8·56 (6) |
| 2 | 46·6±13·56 (7) |
| 3 | 31·8± 6·97 (6) |
| 4 | 33·6± 5·41 (5) |

FIG. 3.—Mean relative spleen weights plus ipsilateral and contralateral lymph node weights of groups of A-strain mice, at death, due to growth of tumour B30.

An analysis of the variance performed on the survival times showed that over the four transplant generations, the decrease in survival times was significant at the 0·1% level (*i.e.* $P > 0{\cdot}001$).

The ipsilateral and contralateral lymph nodes of animals carrying successive transplant generations of B30 were in all cases grossly hypoplastic, whilst the spleens were initially hyperplastic, but became normoplastic as passaging continued (Fig. 3). These changes are in accord with those previously described under similar conditions by Symes (1965*a*).

At the same time there was no significant reduction in the immunocompetence index in animals bearing the first or fourth transplant generations of this tumour for 42 days, or the second generation for 21 days (Table III).

*Histology*

There was no change seen in the appearance of tumour B29 on serial transplantation. In all cases the tumour was poorly differentiated.

With tumour B30, the tumour in the autochthonous host and in the first two transplant generations showed evidence of differentiation into gland acini with ducts. In the third and fourth transplant generations this differentiation was less marked and such ducts as were present showed incomplete epithelial linings.

The host ipsilateral lymph nodes in the third passage of tumour B29, showed hyperplasia in the thymus dependent deep cortical areas.

No evidence of lymph node stimulation was seen in hosts bearing the other passages of tumour B29, or in the animals carrying any passage of B30.

The spleens in animals of the eighth and ninth passages of tumour B29 showed marked hyperplasia of the Malpighian follicles, the splenic white pulp being markedly active in the ninth passage.

### DISCUSSION

The results presented above confirm that on serial subcutaneous passage of A-strain mouse mammary carcinomata in the strain of origin, the killing time of the tumour decreases. Pari passu the immunological response to the tumour involves first the peripheral lymphoid tissue and later, when this becomes exhausted, the spleen. In this connection it is postulated that the spleen functions as a central immunological reserve.

At the same time as these changes are occurring, there is no decline in the immunological competence of spleen cells from the tumour bearing hosts, as assayed by their ability to react against third-party antigens.

It is therefore suggested that the fundamental change involved in the increasing malignancy of the tumour is a change in the tumour cells themselves rather than in the host's immunological response thereto.

This finding is somewhat at variance with the results of others. Linder (1962) found a prolonged survival of skin allografts in mice with spontaneous mammary carcinomata and carcinogen induced sarcomata. In addition Stjernsward (1967) demonstrated that in mice receiving a single injection of 3-methylcholanthrene, there was prolonged depression of the response to weakly antigenic skin allografts during the latent period before a tumour appeared. Stjernsward (1968) also found that following excision of the tumour bearing hind limb of a mouse, treated with 3-MC, this animal supported the growth of a rechallenge with its own tumour better than did a sham amputated isogenic control.

It would therefore seem of interest to repeat the findings reported in the present paper, using a carcinogen induced tumour.

However, that a change in tumour rather than host is involved in the present system is further supported by the following evidence.

(i) Five mammary carcinomata were separately transplanted from the autochthonous host to further isogenic hosts as three subcutaneous transplants in each case. For each tumour a transplant was excised at 14, 28 or 42 days after implantation and transferred to a further host. Tumour growth rate and the survival time of this second host, were respectively directly and inversely proportional to the period for which the tumour was present in the first generation host.

(ii) A mammary carcinoma passaged through two isogenic hosts grew at the same rate on re-transplantation as a tumour maintained for the same time in one host. The tumour maintained in one host was excised and transplanted to the opposite side at the same time as the first tumour line was passaged to the second host.

When this experiment was repeated with a second tumour the line maintained in one host grew faster than that maintained by serial transplantation through three hosts.

These findings will be reported in detail later.

We are grateful to Miss T. W. L. C. Lai for her expert technical assistance. In addition we should like to thank Mr. M. Westwood for his advice on statistical matters, and Messrs. F. E. Badrick and J. Eatough for preparing the illustrations.

The work was supported by a generous grant from the British Empire Cancer Campaign for Research.

### REFERENCES

Denton, P. M. and Symes, M. O.—(1968) *Immunology*, **15**, 371.

Linder, O. E. A.—(1962) *Cancer Res.*, **22**, 380.

Miller, K. A. and Taylor, M. J.—(1948) *Proc. Soc. exp. Biol. Med.*, **68**, 336.

Simonsen, M. (1962) *Prog. Allergy*, **6**, 349.

Stjernsward, J.—(1967) *J. natn. Cancer Inst.*, **38**, 515.—(1968) *J. natn. Cancer Inst.*, **40**, 13.

Symes, M. O. (1965*a*) *Br. J. Cancer*, **19**, 189.—(1965*b*) *Br. J. Cancer*, **19**, 181.

Woodruff, M. F. A. and Symes, M. O.—(1962*a*) *Br. J. Cancer*, **16**, 484.—(1962*b*) *Br. J. Cancer*, **16**, 120.—(1962*c*) *Br. J. Cancer*, **16**, 707.